# New science challenges old notion that mercury dental amalgam is safe

**DOI:** 10.1007/s10534-013-9700-9

**Published:** 2014-01-14

**Authors:** Kristin G. Homme, Janet K. Kern, Boyd E. Haley, David A. Geier, Paul G. King, Lisa K. Sykes, Mark R. Geier

**Affiliations:** 1 International Academy of Oral Medicine and Toxicology, ChampionsGate, FL 33896 USA; 2 Department of Psychiatry, University of Texas Southwestern Medical Center, Dallas, TX USA; 3 Institute of Chronic Illnesses, Inc., Silver Spring, MD USA; 4 Department of Chemistry, University of Kentucky, Lexington, KY USA; 5 Coalition for Mercury-Free Drugs (CoMeD), Silver Spring, MD USA

**Keywords:** Mercury, Dental amalgam, Porphyrins, Chronic mercury toxicity

## Abstract

Mercury dental amalgam has a long history of ostensibly safe use despite its continuous release of mercury vapor. Two key studies known as the Children’s Amalgam Trials are widely cited as evidence of safety. However, four recent reanalyses of one of these trials now suggest harm, particularly to boys with common genetic variants. These and other studies suggest that susceptibility to mercury toxicity differs among individuals based on multiple genes, not all of which have been identified. These studies further suggest that the levels of exposure to mercury vapor from dental amalgams may be unsafe for certain subpopulations. Moreover, a simple comparison of typical exposures versus regulatory safety standards suggests that many people receive unsafe exposures. Chronic mercury toxicity is especially insidious because symptoms are variable and nonspecific, diagnostic tests are often misunderstood, and treatments are speculative at best. Throughout the world, efforts are underway to phase down or eliminate the use of mercury dental amalgam.

## Introduction

Most Americans have dental restorations, and many of those restorations are dental amalgam, known as “silver” fillings. Richardson et al. (2011) estimate that over 180 million Americans carry a total of over one billion restored teeth (based on 2001–2004 population statistics), and that the majority of these restorations are dental amalgam.

Dental amalgam, which contains about 50 % mercury, was once assumed to be inert, meaning that it released no mercury once the filling was placed. Today the US Food and Drug Administration (FDA) and other agencies acknowledge that dental amalgam releases low levels of elemental mercury vapor (FDA 2009). Still debated are the questions of whether these levels are safe, and whether the safety threshold differs among subpopulations (Berlin et al. 2007).

On one side of the debate, the FDA and the American Dental Association (ADA) support amalgam as a safe and effective material for dental restorations (FDA 2013; ADA 2013), and amalgam continues to play a major role in dentistry today (Makhija et al. 2011). However, the safety of mercury dental amalgam is questionable based on recent epidemiological findings.

## Reanalysis of Children’s Amalgam Trial finds harm

Results from the only two randomized, controlled, clinical trials on dental amalgam, known as the Children’s Amalgam Trials, one in New England and one in Portugal, were first reported in 2006 (Bellinger et al. 2006; DeRouen et al. 2006). The New England Children’s Amalgam Trial followed 534 children for 5 years, and the Portugal Children’s Amalgam Trial followed 507 children for 7 years. Both studies found no difference in neurobehavioral outcomes between the amalgam group and the composite (non-amalgam) group—although in both trials the amalgam group showed a statistically significant increase in urinary mercury levels. These two studies, in addition to being widely cited in the literature, are cited by the FDA and the ADA as providing evidence for the safety of amalgam (FDA 2009; ADA 2013). But four recent reanalyses of the Portugal dataset using refined exposure metrics now reveal evidence of harm, as described below. The parent study used a dichotomous exposure metric—amalgam versus composite—which failed to capture the range of exposures within the amalgam group. A 2011 reanalysis used an exposure metric based on amalgam size and years of exposure—and found a significant association between amalgam and the porphyrin biomarkers for mercury-related enzyme blockage (Geier et al. 2011). This association suggests that amalgams are a significant chronic contributor to mercury body burden (Geier et al. 2011).

Using genotype as an independent variable, a 2012 reanalysis by, among others, four authors from the original team, found a highly significant and consistent association between neurobehavioral deficits and an exposure metric derived from annual urinary mercury levels—in boys with a common genetic variant called CPOX4 (Woods et al. 2012). Coproporphyrinogen oxidase (CPOX) is an enzyme on the heme biosynthesis pathway, and the CPOX4 variant has a population frequency of 28 % according to the authors. Of the 23 neurobehavioral tests employed, boys with the genetic variant showed mercury-related deficits in 11 outcomes with a *p* value ≤.05, and 7 of these had a *p* value ≤.01.

A 2013 reanalysis found a significant association between amalgam and a biomarker for kidney damage in the same genetically susceptible subpopulation (Geier et al. 2013). Finally, another 2013 reanalysis found a significant association between neurobehavioral deficits and the exposure metric used in the 2012 reanalysis (derived from annual urinary mercury levels)—in boys with common variants for two metallothionein proteins (Woods et al. 2013). In this paper, Woods et al. explicitly note that the exposure metric reflects exposures from all sources; thus the findings do not support per se the conclusion that amalgams are associated with adverse neurobehavioral effects. However, the parent study (DeRouen et al. 2006) and the corresponding New England study (Bellinger et al. 2006) both found a significant association between this exposure metric and amalgams. Thus, taken as a whole these studies do not support assurances that amalgams are safe; rather they suggest that amalgams may be a significant chronic contributor to mercury body burden, and that this may play a causal role in neurobehavioral deficits and other harm to genetically susceptible subpopulations that are only beginning to be identified.

In the past decade, at least six common genetic variants have been identified that appear to convey increased susceptibility to mercury toxicity from dental amalgam based on epidemiological evidence of clinical harm (Woods et al. 2012, 2013). These genes include: CPOX; brain-derived neurotropic factor (BDNF); 5-hydroxy-tryptamine (serotonin) transporter (5-HTT); catechol O-methyltransferase (COMT), metallothionein MT1M, and metallothionein MT2A (Woods et al. 2012, 2013). In addition, a variant of a glutathione-related gene, glutamyl-cysteine ligase modifier subunit (GCLM), has been associated with higher blood and urine levels of mercury (Custodio et al. 2005). (The glutathione system comprises the major detoxification mechanism for mercury.) Moreover, a recent study of methylmercury exposure from dietary fish also found that a variant of GCLM, as well as a variant of another glutathione-related gene, glutathione-*S*-transferase modifier subunit (GSTM), are associated with altered mercury concentrations and antioxidant status in humans (Barcelos et al. 2013).

In theory, many more susceptibility genes are likely—including the ApoE4 allele implicated in Alzheimer’s disease (Mutter et al. 2010)—because mercury blocks sulfur groups within proteins, which are coded by genes that vary among individuals (Berlin et al. 2007). Indeed, many glutathione-related enzymes are highly polymorphic (Barcelos et al. 2013).

## Mercury’s insidious toxicity

Mercury’s toxic mechanism is broad. It binds sulfur, which is ubiquitous in cellular proteins, both structural and functional (ATSDR 1999; Berlin et al. 2007; Kern et al. 2013). For example, it blocks the sulfhydryl active sites within enzymes, receptors, signaling molecules, and membrane transport channels (Berlin et al.^2007^). These mechanisms disrupt key cellular processes in ways that depend on genetic individuality and on micronutrient status (Berlin et al. 2007). Effects include altered membrane permeability, increased oxidative stress, peroxidation of lipid membranes, mitochondrial dysfunction, and altered production of neurotransmitters, cytokines, and hormones (ATSDR 1999; Berlin et al. 2007). The resulting symptoms—variable and nonspecific—may be difficult to detect until much damage has been done.

Animal studies reveal that mercury vapor from amalgam is rapidly absorbed and distributed throughout the body, concentrating in organs—including those of the fetus (Lorscheider et al. 1995). Animal and human studies reveal that mercury is transferred to breast milk in proportion to maternal dental amalgam load (Richardson et al. 2011; Lorscheider et al. 1995). Once in the body, some mercury is eliminated in urine and feces, but evidence suggests that elimination slows as detoxification enzymes become impaired, yielding increasing retention and unpredictable toxicity (Mutter et al. 2007, 2004).

The developing neuron is the most sensitive target for mercury (Berlin et al. 2007). Studies on neurons in culture find growth impairment at the same mercury concentrations that are found in neonatal infants of amalgam-bearing mothers with no other known exposures (Berlin et al. 2007; Kern et al. 2012).

Mercury’s broad toxic mechanism and the resulting nonspecific symptoms, as well as the diagnostic difficulties described below, make chronic mercury toxicity difficult to study in humans. Indeed, many epidemiological studies have failed to find evidence of a clear association between amalgam and clinical health effects. Yet many of these studies use insufficient time-frames as well as flawed measures of exposure such as blood or urine levels as described below (Mutter et al. 2007, 2004). In addition, none appear to consider genetic susceptibilities. Thus few conclusions can be drawn.

## Diagnostic difficulties

Chronic mercury poisoning is described in the toxicology literature but is not yet recognized by most physicians or institutions. Medical textbooks give the issue little coverage [for example, Fauci (2008)], so the typical, slow onset of nonspecific symptoms is likely to be misdiagnosed.

No reliable diagnostic test exists for chronic mercury poisoning (Berlin et al. 2007; Mutter et al. 2007). A porphyrins panel can reveal the footprint unique to many toxic metals including mercury, but since porphyrins are easily destroyed (Woods 2009), the risk of false negatives is high.

Current medical diagnostic criteria target acute rather than chronic poisoning [for example, Fauci (2008)]. Such criteria often require a finding of elevated blood or urine mercury levels [for example, Goldman and Schafer (2011)] even though these media reveal only recent exposures and do not reflect body burden or symptoms (Berlin et al. 2007; Mutter et al. 2007). Counterintuitively, some individuals with a high body burden may show low mercury levels in blood, urine, hair and nails—apparently due to impaired excretion, which yields increased retention (Mutter et al. 2007, 2004; Lorscheider et al. 1995).

Not only is diagnosis difficult, but treatments are controversial and are speculative at best. Even removal of amalgam is problematic, causing high exposures to respirable amalgam particulate matter as well as to mercury vapor (Richardson 2003).

## Unsafe exposures

A simple risk assessment—a comparison of estimated exposures versus regulatory safety standards—yields cause for concern. The World Health Organization estimates that the typical absorbed dose of mercury from amalgams is 1–22 micrograms per day (μg/d), with most people incurring doses of less than 5 μg/d (IPCS 2003). Considerable variation exists, with an upper range of ~100 μg/d associated with gum chewing (Berlin et al. 2007). Exposure variables include the total amalgam surface area, the physical and chemical composition of the amalgam, the mechanical stresses of chewing and bruxism, the proximity to other metals, and the oral conditions of temperature, pH, and negative air pressure. The FDA assumes an exposure of 1–5 μg/d in its current amalgam rule [PHS 1993 (as cited in FDA 2009)].

Regarding safety standards, the US Environmental Protection Agency (EPA) provides a reference concentration (RfC) for chronic mercury inhalation of 0.3 μg/m^3^, which was set in 1995 (EPA 1995). As shown in Table 1, this standard can be converted to a tolerable daily exposure of 4.9 μg/d—and this value is virtually the same as the FDA’s assumption for typical amalgam exposure of up to 5 μg/d. In other words, people with typical amalgam exposures are at the regulatory safety threshold with no margin of safety, and people with above-average exposures exceed the safe threshold.

**Table 1:** Amalgam exposures versus regulatory safety standards

Exposure estimates for mercury vapor from amalgam (μg/d)		Safety standards for chronic mercury vapor inhalation	
		California EPA REL (reference exposure level) (2008) 0.03 μg/m^3^, converted to μg/d	US EPA RfC (reference concentration) (1995) 0.3 μg/m^3^, converted to μg/d
Estimated typical chronic intake from amalgam (FDA; ATSDR)	1–5	0.5^a^	4.9^a^
Estimated range of chronic intake from amalgam (WHO; ATSDR)	1–22		
High-end chronic intake from amalgam (ATSDR)	~100		

The middle to upper ranges of exposures to mercury vapor from amalgam exceed the US EPA safety standard for chronic mercury inhalation. The tighter California EPA standard appears to preclude any amalgam fillings

^a^Assuming a ventilation rate of 16.2 m^3^/d (US EPA)

The FDA acknowledges the absence of a margin of safety but notes that the EPA’s regulatory standard includes an uncertainty factor that was derived to be protective (FDA 2009). Yet this uncertainty factor is tenfold more lenient than that used by the California Environmental Protection Agency (CalEPA) as described below. The FDA also claims that the levels of exposure from amalgams are well below levels actually known to cause adverse effects (FDA 2009) even though these data are gleaned from occupational studies of healthy workers and are not intended to apply to the general population. The FDA also notes that amalgam is a commonly used device with a low frequency of adverse events reported to the agency (FDA 2009).

As shown in Table 1, almost no amount of amalgam is safe under the CalEPA standard—the reference exposure level (REL) for chronic mercury inhalation of 0.03 μg/m^3^—which was set in 2008, and which includes an uncertainty factor that considers developmental toxicities (CalEPA 2008). Richardson et al. (2011) estimate that 122.3 million Americans exceed the mercury dose associated with the CalEPA standard, and 67.2 million Americans exceed the more lenient US EPA standard.

## Public policy

In use since the 1800s, dental amalgam has never undergone the regulatory proof-of-safety testing that is required for other medical implants under US law. Under the 1976 Amendments to the Federal Food, Drug, and Cosmetics Act, Congress directed the FDA to assess the safety of medical and dental devices and to require premarket approval of safety for any device that “is intended to be implanted in the human body” (USC §§ 360a, et seq.), yet the FDA has interpreted the statute to exempt dental amalgam.

Norway and Sweden have banned dental amalgam (Norway Ministry of Environment 2007; Sweden Ministry of Environment 2009). Germany and Canada advise against its use in pregnant women and children (PHS 1997). According to the largest mercury-free professional dental association, there is no situation in which amalgam is either required or preferred (IAOMT 2013). The predominant alternative—resin-based composite—requires more skill to install, but when properly placed it is as durable as amalgam according to a recent meta-analysis (Heintze and Rousson 2012).

Concerns exist regarding the ingredient in resin-based composite called bisphenol A (BPA), which is under investigation as an endocrine disruptor. But dental restorations appear to be a minor source relative to other environmental exposures such as food packaging (NIEHS 2013). A 2010 World Health Organization report found that BPA from dental materials is unlikely to contribute substantially to chronic exposure (Bailey and Hoekstra 2010).

In October 2013, over 140 nations signed the Minamata Convention, a set of legally binding measures to curb mercury pollution that was forged over the past 4 years by the United Nations Environment Programme (UNEP 2013). Participants agreed to phase down the use of dental amalgam via a menu of strategies to discourage amalgam use and promote alternatives.

Meanwhile, the US FDA has stated that it is actively reviewing the safety of amalgam but has made no further comment on the issue since 2010. If the White House initiative to restore scientific integrity to federal policymaking is successful, the US may soon join the other nations that have banned or restricted mercury dental amalgam.
